# Supplementary material for: Multimodal imaging and functional analysis of the chick NMDA retinal damage model
Source: PLoS One. 2021 Sep 7;16(9):e0257148. doi: 10.1371/journal.pone.0257148 (PMC8423281; doi:10.1371/journal.pone.0257148)
Supplement: S1 File — (ZIP) [file pone.0257148.s004.zip › SD-OCT/C200727/012/D15-PWk3/C200727-012_OS_R_14_0_REGAVG0000049/Manual_PreDefineThreeByFourGridRadialRetina_1.pdf]

Three By Four Grid Radial Retina Template Report

Patient ID	C200727	Name	See InVivoVue
Exam Date	See InVivoVue	Exam Time	See InVivoVue
Eye	OS	Averaged	N/A
Depth (mm)	1.65	Depth Samples	1024
Pitch (um/pix)	14.000 x 2333.333 x 1.608	Refractive Index	See InVivoVue
Scan Type	Radial 14.000mm x 14.000mm x 1.647mm ,<br>1000 x 6 x 1024		

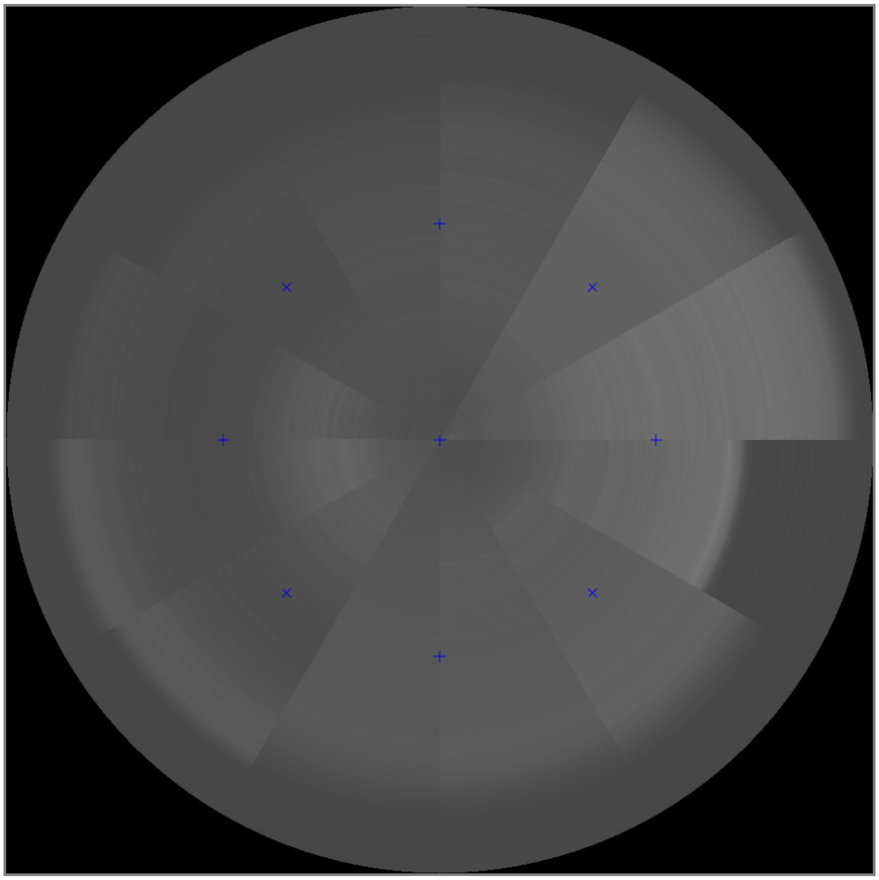

Thickness Metrics

Analysis	Average(mm)	Minimum(mm)	Maximum(mm)	STDEV(mm)
IRNFL - RPE	0.2747	0.2634	0.2902	0.0085
IRNFL - ORNFL	0.0376	0.0187	0.0693	0.0173
ORNFL - OPL	0.1592	0.1404	0.1866	0.0141
OPL - ETPRS	0.0589	0.0506	0.0649	0.0046

Q:\Bioptigen\Data\images\site3000254\image\2020.08.17\C200727-012_OS_R_14_0_REGAVG0000049.OCT

1/8/2021 3:04:56 PM
